# Supplementary figures and images for: Temporal transcriptomic profiling of pulmonary thromboembolism reveals persistent NETosis- and ferroptosis-associated gene signatures and enhanced thrombolysis with adjunctive DNase I
Source: PLoS One. 2026 May 29;21(5):e0349853. doi: 10.1371/journal.pone.0349853 (PMC13221060; doi:10.1371/journal.pone.0349853)

Top 20 Important Genes

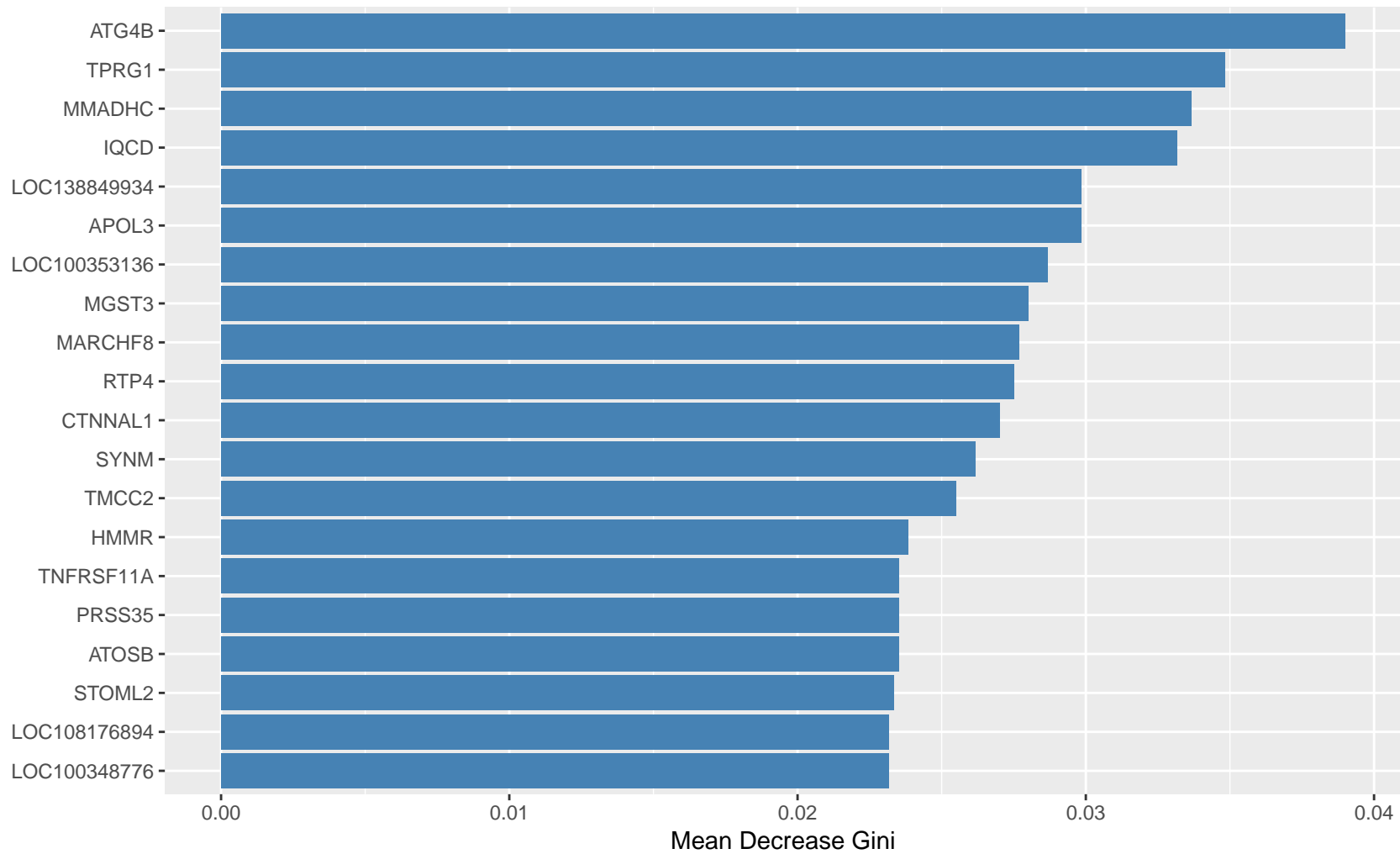

Supplement: S1 File — (ZIP) [file pone.0349853.s001.zip › S1_File/D1 VS C.pdf]

Top 20 Important Genes (Random Forest)

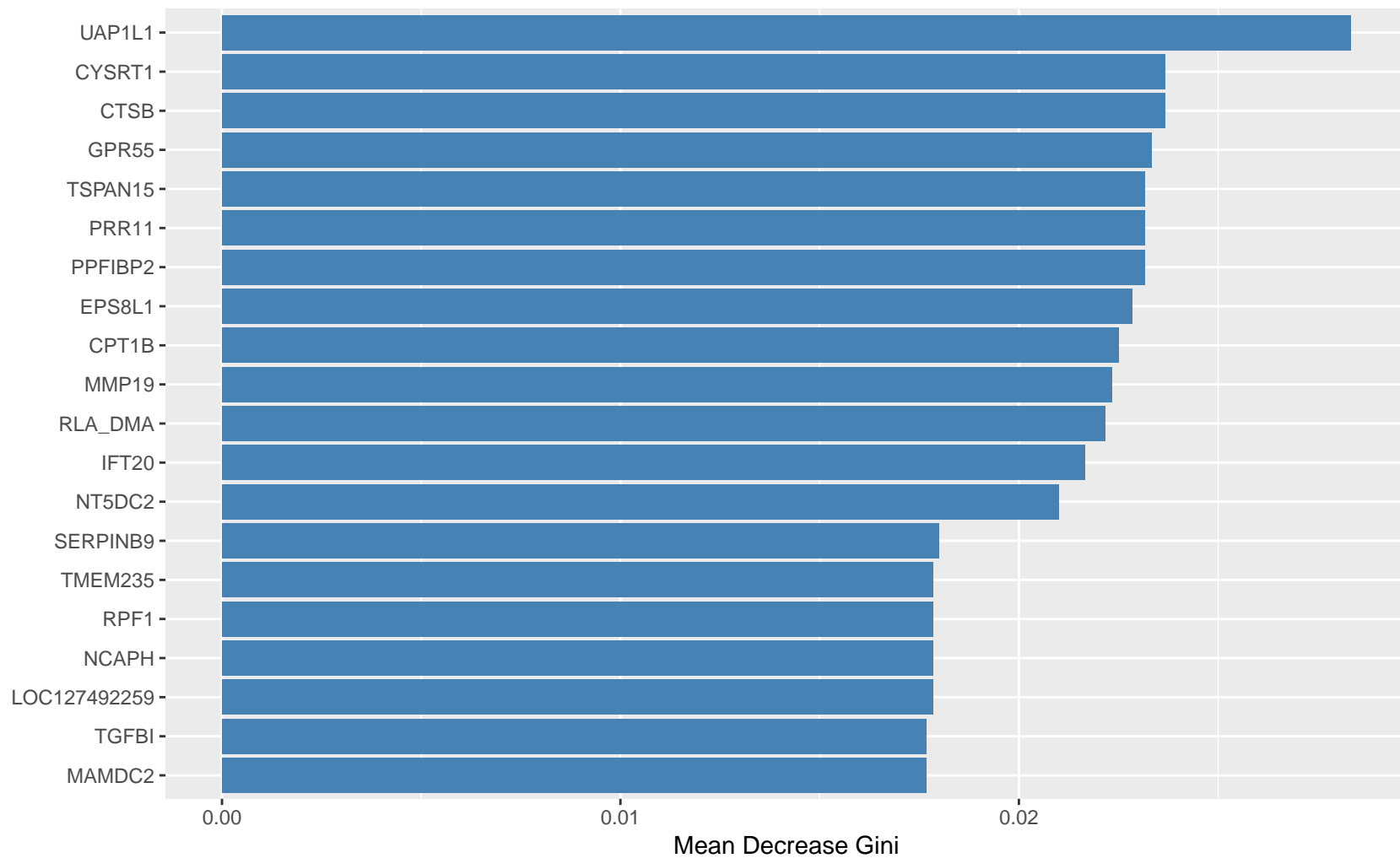

Supplement: S1 File — (ZIP) [file pone.0349853.s001.zip › S1_File/D14 VS C.pdf]

Top 20 Important Genes (Random Forest)

Gene

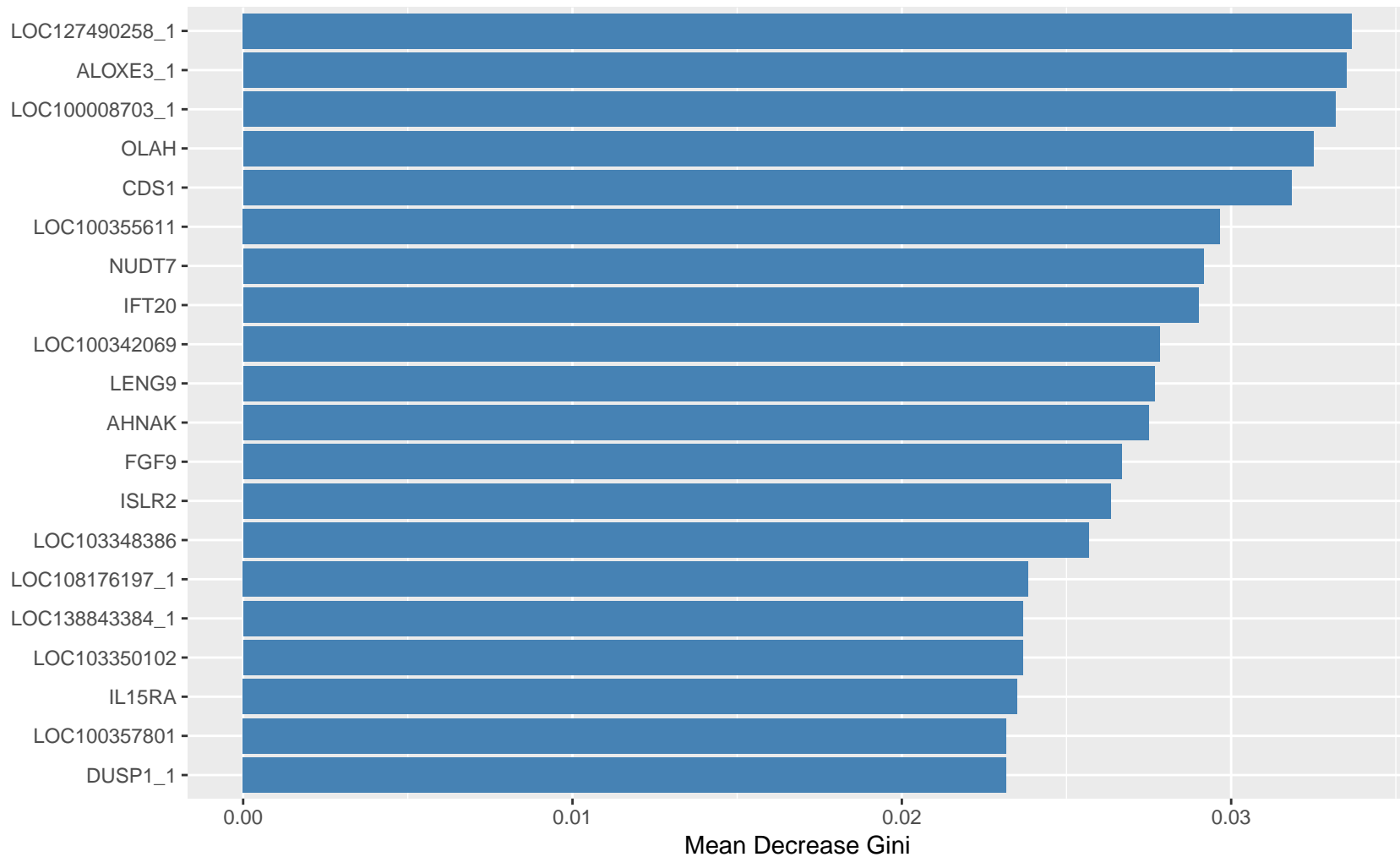

Supplement: S1 File — (ZIP) [file pone.0349853.s001.zip › S1_File/D3 VS C.pdf]

Top 20 Important Genes (Random Forest)

Gene

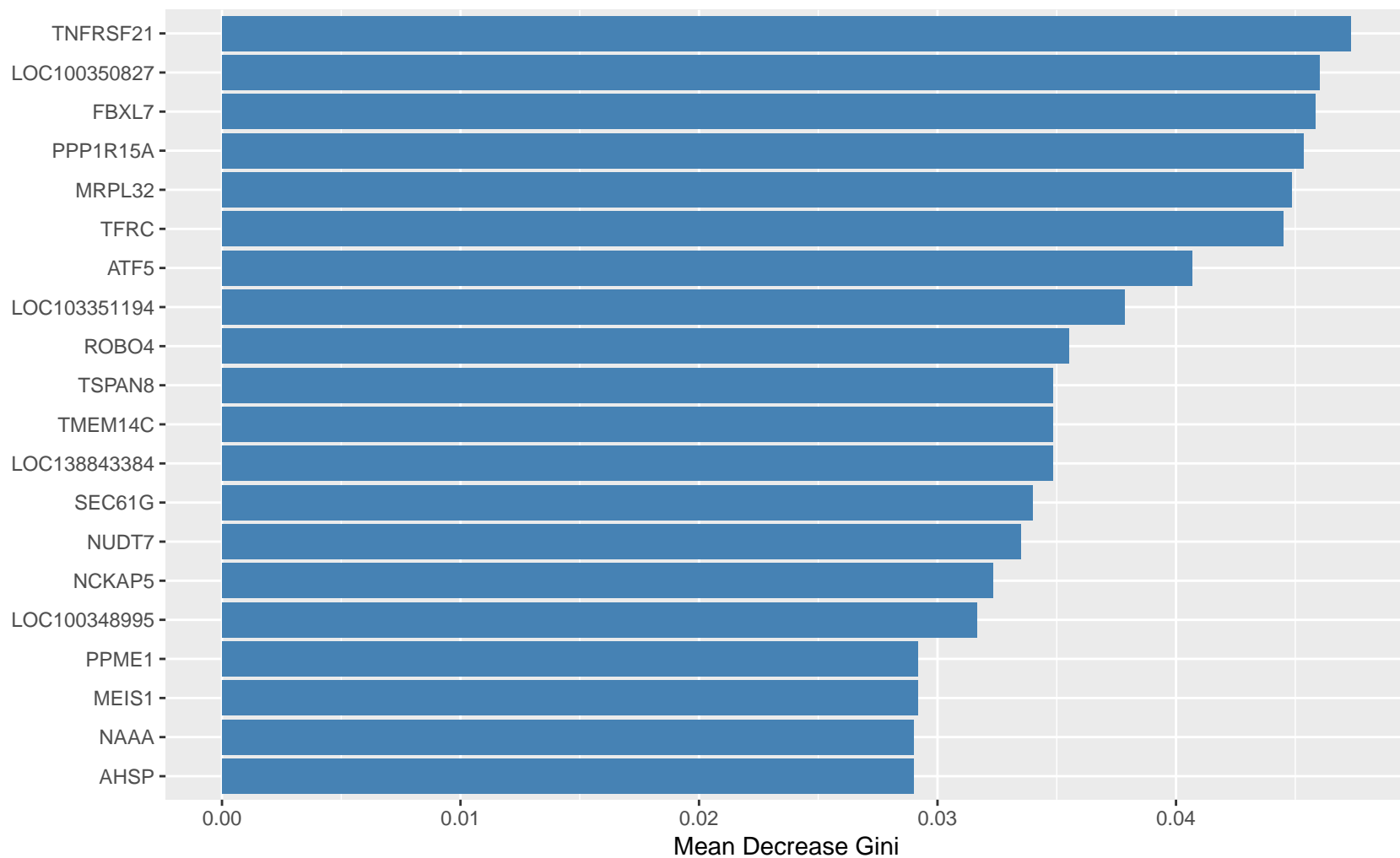

Supplement: S1 File — (ZIP) [file pone.0349853.s001.zip › S1_File/D7 VS C.pdf]
