# Supplementary material for: Temporal transcriptomic profiling of pulmonary thromboembolism reveals persistent NETosis- and ferroptosis-associated gene signatures and enhanced thrombolysis with adjunctive DNase I
Source: PLoS One. 2026 May 29;21(5):e0349853. doi: 10.1371/journal.pone.0349853 (PMC13221060; doi:10.1371/journal.pone.0349853)
Supplement: S1 Text — (DOCX) [file pone.0349853.s002.docx]

**S100A8 XM_002715343.5 CDS：353-622**

atgccgactgatctggaaaattccttgaactccatcatttccgtctaccacaagtactccctggagaaaggaaattaccacgccctctacggggatgatctgaagaagctcctagctactgagtgtcctcagtacacgaagaaaaaggatgcagacacttggttcaaagagctggatatcaacagtgatggggccatcaacttccaggagttcctcatactgattgtgaagataggcgtgtccgcccacgaagacagccacaaggcgtag

**S100A9 NM_001256473.1 CDS：40-438**

atgtcctgcgggatgtcgcagctggaacgcagcatcgataccatcatcaacgtcttccaccagtactccrtgcgggtggggccgcgggacagcctgagccagaaggaattcaagcagctggtacagaaagagctgcacaacttcctcaagaaggaggcgagggatgagaaagccataaatgatatcatggaggacctggacacgaaccaggacaagcagctgagctttgaagagttcgtcatcctgatggcaaggctggtccacgcctcccacgaggagatgcacaagaatgccccccatgaccatgagggccacagccacggcccaggccttggcgggggtggtcctggccatggacatgggcatagtcacggccatggccatggccacagccactaa

**LCN2 XM_002722973.5 CDS：99-695**

atgcctcttggtctcctgtggctgggcctcaccctgctgggggccctgcacatccaggcccaggaccccaccccaaaactgatccctgccccatctctgcgcagggttcccctgcagcggaacttccaggatgaacagttccagggcaagtggtacgtcgtgggcctggcgggcaatgccgtccagaagagggaagaaggccaggagccgatgtacagcaccacctacgagctgaacgaagatcgcagcttcaacgtcacctccaccttgctcagggaccagcgctgtgaccactggatcaggactttcgtccccacgtcccggcccggccagtataacctgggcaacatcaagagttaccctggggtgaagaactacattgtgcgcgtggtggccaccgactacagccagtatgccatgatgttcttcaggaagggttccagaaacaagcaattcttcaagaccaccctctacgggagaaccaaggagctgagccccgagctgagggagaggttcacccgcttcgccaaatctctgggcctccccgacgaccgcatcgtcttccccacccccatcgaccagtgcattgatgactga

**LTF XM_008260521.3 CDS：38-2164**

atgaggctcctcttccccaccctgctgttcctgggggtcctgggactgtgtccggctgcgcccaggaagagcgttcggtggtgcaccaagtccccgccagagacagccaaatgctccaggctgcggaagaacatgagaaagcaaggcggcccgccgctgagctgcatccggaagccgaccgccctggggtgcatccaggccatcgcgggaggcaaggcggatgccatgaccctggatagcggcttggtgttccaagccagccaggccccctacaaactgcgacctgtcgccgtggaggtctacgggacagaagcgaagccacagacccactactatgccgtggccgtggccaggaagggcagcagcttccagctgaaccagctccggggccggaagtcctgccacacgggctttggccggactgctggctggaacatccccatagggacgcttcgcccgttcttgaactggacggggccccccaagccccttagtgctgctgtggccgagttcttctccagcagctgtgtgcccggggcagacggacagcgcttccccaacctgtgcaagctgtgtgtagggaggggcccaaaccactgtgccttctcctccctggagccatactttggctactccggcgccttcaggtgtctgagagacggggccggagatgtggctttcatcagggagactacggtgtttgaggacctgccggacaaggccgcaagggacgcgttcgaactgctctgcccggacaacacccggaggccggtggaccagtacgagcggtgccacctggcccgagtgccttcccacgctgttgtggcccgcagtgtcaatggcagggaagatgccatctgggagtttcttcgccaggcgcaggaaaggtttgggaagaacaagtcgaagtcgttccagctcttcggctcccccgcagggcagaaggacctgctgttcaaggactccgccatcgggttcctgagggtccccgagaagatagacgctgggctgtaccttggctacagctacgtcactgccatccagaacctggggaaaacggagatggccgtggcagcccggcagtccctggtcgtgtggtgcgccgtgggcagcaaggagcagcacaagtgcagccagtggagccgcctgagccagggcagcgtggcctgtgccgtggcacccaccacggaggactgcatcgccctcatcctgaagggagacgctgacgccatgagtttggatggaggatacatctacaccgcgggcaagtgtgggctggtgcccgtcctggcggagaattcaatatcccgagaaggtgtcagctctgactgtgtgaacagacgatcggaagggtatcttgccgtggcggtggtgaggaaatcggaccctgacatcacctggaactctctgaggggcaggaagtcctgccatacggccgtgggcaggaccgcgggctggaacatccctgtgggcttgctcttcaaccagacaggctcctgcaggttcgatgaattcttcagtcagagctgcgcccccggctctgacccgagatccagactctgcgctctgtgtgttggtaacgagaggggcgaggagaagtgtgtgcccaacggcaacgagaggtactacggctacaacggcgctttcagatgcctggctgaaaatgctggagatgttgcattcataaaagctgtcactgtcttgcagaacaccaacgggaagaacccggagccctgggccaggcacttgaaacaggaggattttgagctgctgtgcctggacggcacccggcggcctgtggccgaggccaagaactgccacctggccatggcccccagccacgcggtggtgtcgcgcaaggacaaggtggagcgcctggagcaggtgctgctggagcagcaggctaagtttggaaagaatggagccaggtgcctgggagagttctgcttgttcaagtccgattccccaaaccttctgttcaatgacaacacggagtgtctggccaggctccaaggcagaacaacgtatgaaaaatatttgggaccccagtatgtggcagccattggtcatctgagacgctgctcgagctctccgctcctggaagcctgcgccttccttaggaagtaa

**MGST3 NM_001082138.1 CDS：5-463**

atggctgtcctctctaaggaatacggtttcgtgcttctgacgggtgctgccagctttgtaatggtgcttcacctcgccatcaacgttaacaaggctcgcaagaagtacaaagtggagtatcctgttatgtacagcacggaccccgaaaatgggcacctcttcaactgcattcagcgagcccaccagaacacgttggaagtgtaccctcccttcttgttttttctggctgttggaggtgtttaccacccacgtatagtctctggcttgggctgggtctggatcgttgggcgagttctttacgcttatggctattacacaggagaccccagcaagcggtaccgaggagccgtgtcctccctggccctcttcggcttgatgggcaccacggtgtgctctgctttccagcatctcggctggattaagcccaggttgggcagtggggccaagccctgccattaa

**APOL3 XM_002711435.4 CDS：228-1256**

atggacccagaggacgaaagctccatcgcccacatcgtcaggtatttccaggacgaggtgagcagagaggacctgcagctcctgctgacggaagacgaagcctggcagatattcgtgaaggaggcggagttgtccggggatgaggcagagtcgctgcgtgatgctctgacggagtttgcgacagacgtggacttggacgtggaggacaaagtcgcgctccagaagtacctgctggaaagggagaggtttttgaacgagtttcctcaggcgaaagccgagctggaggagtgcatagcaaagctgcacgcccttgcagataaggttgaccaggtgcaccggaactgcaccatctccaacgtggtggccaccagtgctggcgctgtctctggcatcctgaccatcgctggcctggctctggcccctgtgacagtaggggccagtctggcgctcacagcaacgggattggggctggggacagcagctgcggtgaccggagtttccaccagcattgtggagcacgctaccaccatctcagccgaagccgaagccggccgcctgacgtcgaccagcgtcaagaaggtggagaaggtggccgaggccgtgggtcagagcgtgcccaaagtcattgccataaccaagtcttgcatccaagtcctgcaaggcatcgggaagaatgtccgtgccctcaagatagcccagaccaactcccgcttagtggccagcgccaagcgcctcatgaccactgggaaaatctcagcccggagcagcaggcaggtccagagagcctttggcggcaccgcactggcaatgagcaagggagcccgcatgatgggtttcgccaccgcaggcatcttccttctgatggacgtggtcaccctggtgaaagagtcgcagcacctgcacgagggggccaagtccaagtcggctgaggagctgagggagcgggcccaggagctggccaggaagctgcaggagctcacggagatccatagaagcctgcagccggacccaatgcagtggatgctgtga

**OLAH XM_008268328.3 CDS：293-1090**

atggagagaagagagcaagctgggagaaccaggaatgaaagagttttgaactgcatataccaaaatcctaatgcaatttttaagctgatttgctttccctgggcaggaggtggctccacttattttgccaaatggggccaaaaaattcatgactcactggaagtgcactctgtaaggcttgctggaagagagagcagatttgaagaaccttttgcaagcgacatgtaccagatagtggatgaaatcgtttgtgctctgctgccaatcatccaggataagccatttgcattttttggccacagcatgggatcctatattgctttcatgacggcactgtacctaaaagaaaatcacaagctagaaccgatgcacttcttcgtgtcaagtacaactcctcctcattccaaagacaggtttcaagttcccaaaagtaacgagctgtcagaagaggagctgagccggtatcttgtggacttcggcggcactccccaggactgccttgacgacaaggagtttttccagcaatacgctcccgtgctgctggcggatgctgacattatgcgtaattacacctttgacgcaccctccaaggctgtcatttcttgtgacttaacatgtttcctgggctctgatgatattgcaaaggatatgcaagcctggaaagacataaccagtggaagtcttgacgttcatgtgcttcctggagaccacttttatcttaaggaacctgccaatgagaatttcatcaagaactacatagccaagtgtctggaactatcgtcgcttactaattgttag

**ATF5 XM_051841677.2 CDS：246-1061**

atgtcactgctggcgaccctggggctggagctggacagggccctgctcccagctagcgggctgggctggctcgtcgactatgggaagctccccctggcccccgcccccctgggcccctatgaggtcctggggggagccctggagggcgggcttccagggggaggagagcccttggcaggggacggcttctcggactggatgacggagcgggtagacttcaccgccctcctccctctggagcccaccgtgcccccaggcgccctgcccccgccctccccctccgcccctgacctggaagccatggcctccctgctcaagaaggagctggagcagatggaggacttcttcctcgaggccccgctgctcccggccccctccccgccgccgcccgccctccccctccccgtccccagctttgacctcccccagccccctgccctggacaccctggacttgctggccatctactgccgcggcgaggccggccagggggattcgggcttggtgcccccgcccccgccgccgcagaccccgcccccgcccccagcccctcgcccggccccttaccccagtcctgccacggcccgaggggaccgcaagcagaagaagagagaccagaacaagtccgcggctctgaggtaccggcagaggaagcgggcagagggcgaggccctggagggcgagtgccaggggctggaggcgcggaaccgggagctgagggagcgggccgagtcggtggagagggagatccagtacgtcaaggacctgctcatcgaggtgtacaaggcgcggagccagaggggccgcagcagctag

**TSPAN15 XM_051823853.2 CDS：137..886**

atgccccgcggggactcggagcaggtgcgctactgcgctcgcttctcttacctctggctcaagttctcgctcatcatctactccacggtgttctggctcatcgggggcctggtcctgtccgtggggatctacgcggaggtcgagcggcagaaatacaagaccctggaaagtgccttcctggctcctgccatcctcctcctcctgctgggggtcatcatgttcatcgtctccttcatcggtgtgctggcctccctgcgggacaacctgtgcctcctccaggcgtttatgtacatcctcgggatctgcctcatcatggagctcattggcggcgtgatggccttgatcttccggaaccagaccattgagtttctcaacgacaacatcagaagaggaattgagaattactatgatgacctggacttcaaaaacatcatggactttgttcagaagcagttcaagtgctgtggcggcgaggactaccgagattggagccagaaccagtaccacgactgcaatgcccccgggcccctggcctgcggggtgccctacacctgctgcttcagaaacacgatgtcgtcaacaccatgtgtggctacaaaaccatcgacaaggagcgcctcagcgtgcaggacgtcatctacgtgcggggctgcaccaatgccgtgctcatctggttcatggacaactacaccatcatggcgggcctcctgctgggcatcctgctcccccagttcctgggggtgctgctga

**CYSRT1 XM_070059668.1 CDS：1671-2105**

atggacccccacgagatggttgtgaagaacccgttcgcccacatcagcatcccccgggcccacctgcggccggccctggggcagcagctggaggccgcgccctgctcgacatcctcagtgacacagccactgcccgaggggccctgtgccccccggccggccagccctctgcagcacccccagactctggggcccaagggggcccaaggggccaaggggaccccgggggtggccctcggccagagccagcaggcttggcagcagcccagcaacccctatggccccgggcagcgcccggcgggactgacctatgctggcctgccccccgtggggcgtggcgacgacatcgcccaccactgctgctgctgcccctgctgctcctgctgccactgcccccgcttctgccgctgccacagctgctgcgtcgtctcctag
