# Supplementary material for: Temporal transcriptomic profiling of pulmonary thromboembolism reveals persistent NETosis- and ferroptosis-associated gene signatures and enhanced thrombolysis with adjunctive DNase I
Source: PLoS One. 2026 May 29;21(5):e0349853. doi: 10.1371/journal.pone.0349853 (PMC13221060; doi:10.1371/journal.pone.0349853)
Supplement: Attachment — Submitted filename: Comments to the Authors.pdf [file pone.0349853.s004.pdf]

Comments to the Authors

General Assessment

The study addresses an important clinical problem and combines multiple experimental layers to explore temporal changes after PTE. The manuscript is very well written and connect the main ideas properly. The experimental design and methodology -including techniques- are in general extensive and robust. However, the manuscript require certain major revisions. Below, I provide detailed comments of specific subjects and comments by section.

Major revisions

1. **Whole-Blood RNA-seq and Cell-Type Attribution:** The manuscript repeatedly attributes findings to neutrophils, yet RNA-seq was performed on whole blood and changes in gene expression may reflect shifts in leukocyte proportions, not being transcriptional regulation within neutrophils. For example in a recent proteomic studies authors found substantial markers hidden in bulk analysis in comparison to a group -specific cells (below)

Ryu T, Kim SY, Thuraisamy T, Shin J, Jang Y, Kam TI, Na CH. In-Depth Cell-Type-Specific Proteome Landscape of the Brain from Human Amyloid-β Overexpression Mouse Model. Adv Sci (Weinh). 2025 May;12(20):e2409318. doi: 10.1002/advs.202409318. Epub 2025 May 8. PMID: 40345165; PMCID: PMC12120748.

2. **Cross-validation of NETs/ferroptosis proteins**: Besides proteins were validated though a second technique method, personally I consider it was not the best choice due to both methods (qPCR and transcriptomics) work on the RNA amplification (technically cDNA). I strongly suggest validating these or others relevant proteins through a direct protein approach. Also, I consider that should highlight the limitations of working with transcriptomics and extrapolate this in neutrophils (in this case a mix of leukocytes, which is more complex to justify) because the neutrophil is a terminal-differentiated cell with poor transcriptomics/proteomics correlation (below)

Rørvig, S., et al., Proteome profiling of human neutrophil granule subsets, secretory vesicles, and cell membrane: correlation with transcriptome profiling of neutrophil precursors. Journal of Leukocyte Biology, 2013/10/01. 94(4).

Hoogendijk, A.J., et al., Dynamic Transcriptome-Proteome Correlation Networks Reveal Human Myeloid Differentiation and Neutrophil-Specific Programming. Cell Reports, 2019/11/19. 29(8).

3. **NETs/Ferroptosis axis:** During results you mention LTF and LCN2 as NETs/ferroptosis-related genes both that definition is too ambiguous due those genes are also related to process of general activation, degranulation, among others which may imply or not NETosis due to those proteins are within the secondary granules and will be secreted even before the NETs release. The data show co-occurrence of NET-associated markers (e.g., MPO-DNA, S100A8/A9) and iron-related/oxidative pathways, but no direct evidence of ferroptotic cell death is provided. Avoid the use of ferroptosis directly to fibrosis unless further mechanistic evidence is added..
4. **Therapeutic (rt-PA ± DNase I):** DNase I is described as intravenous in Methods but nebulized in parts of the Abstract, this inconsistency must be corrected. In these results was observed that the use of DNAse may improve the majority of the measured parameters, although the stadistics comparing the treatments must be done in the same graph (example in the first column, figure 6) the statistics between rt-PA vs rt-PA/DNAse should not be done with the differences (as I understood what you did), please clarify this. Additionally, the effect of DNAse I alone is required and important to observe whether has the same effect as rt-PA, has zero effect or if its addition makes a synergic effect with rt-PA.

Section-Specific Comments

Introduction

- Well written and clinically grounded. I suggest explaining briefly the concepts and mechanisms of NETs, ferroptosis and how both are connected.

Graphs

- In the caption of figures should be described meaning of colors, abbreviations. Statistics and tests applied for that graph/panel (if apply). Place centered titles over the graphs indicating the comparisons -for example figure 3- it would improve considerably the reading.
- Replace * for p-values and add the individual replicates (as dots), those changes will give more credibility for general view.
- If possible, increase the size of title and distribute homogeneously the graphs so avoid blank spaces and figures can be bigger.

Results

- Descriptive and comprehensive. I suggest explaining very briefly the meaning of the measured proteins, staining or parameters when is mentioned the first time -currently many are mentioned during discussion-, so it would improve smooth and quicker reading.

- Describe of terms related segmented by type of enrichment (CC, BP, MF). These enrichments were considered in all DEGs, or only for upregulated? Or downregulated? Clarify this. Which background of genes were used for enrichment?

- From where was the list of top NETs, ferroptosis related genes extracted?

Discussion

- Thoughtful and well structured. During the discussion all the proteins meanings are explained and discussed, I suggest the meaning explanation should appear once is mentioned during introduction and results.

- Which advantages, and limitations the experimental model applied in this study in comparison to other *in vivo* models for the same disease (or some condition related).

- Speculative sections (especially on ferroptosis and self-reinforcing inflammatory loops) should be clearly labeled as **hypotheses**, not conclusions.
