# Supplementary material for: Temporal transcriptomic profiling of pulmonary thromboembolism reveals persistent NETosis- and ferroptosis-associated gene signatures and enhanced thrombolysis with adjunctive DNase I
Source: PLoS One. 2026 May 29;21(5):e0349853. doi: 10.1371/journal.pone.0349853 (PMC13221060; doi:10.1371/journal.pone.0349853)
Supplement: Attachment — Submitted filename: response to reviewer.docx [file pone.0349853.s005.docx]

**Response to Reviewers**

Dear Editor and Reviewers,

We sincerely thank you for the careful evaluation of our manuscript and for the constructive and insightful comments. We have revised the manuscript extensively in response to these concerns. In this revision, our goal was not to overextend the available data, but to strengthen orthogonal support where feasible, clarify the limitations of whole-blood transcriptomics, and align all conclusions with the actual level of evidence provided.

The major revisions include:
(1) addition of a new independent four-group pharmacological validation experiment (Control, PTE, PTE + DNase I, and PTE + ferrostatin-1 [Fer-1]);
(2) substantial moderation of mechanistic and translational claims throughout the manuscript;
(3) clearer acknowledgment of the limitations of whole-blood RNA-seq for cell-type attribution;
(4) revision of the statistical description and figure legends; and
(5) clarification of the therapeutic intervention design and its interpretive limitations.

Below we respond point by point.

### 1. Editorial concern: the manuscript overinterprets associative findings and requires stronger mechanistic support.

**Response:**
We agree with this important concern and have revised the manuscript extensively to avoid overinterpretation of associative data. Throughout the revised manuscript, we now use more cautious language and explicitly distinguish descriptive observations from mechanistic inference. We have removed or softened statements that could imply definitive proof of a mechanistic “axis” or direct causal interaction.

To strengthen mechanistic support where feasible, we performed a new independent four-group pharmacological validation experiment at day 7 post-PTE (Control, PTE, PTE + DNase I, and PTE + Fer-1; n = 5/group). These new data are presented as **Figure 7** and incorporated into the revised Abstract, Materials and Methods, Results, Discussion, and Conclusion. The new experiment provides additional **pharmacological support consistent with** the involvement of NETs-associated signaling and ferroptosis-related injury processes in PTE, but we now explicitly state that these data do **not** constitute definitive proof of an in situ mechanistic pathway.

Accordingly, the revised manuscript now frames the proposed NETs–TLR9–NF-kB/GPX4 link as a **supported but not definitively proven** mechanism.

### 2. Please add a causal intervention targeting ferroptosis or NETs independently.

**Response:**
We appreciate this suggestion and addressed it directly in the revised manuscript by adding an independent pharmacological validation cohort. In this experiment, DNase I was used as an upstream intervention targeting NETs-associated extracellular DNA, whereas ferrostatin-1 (Fer-1) was used as a ferroptosis inhibitor.

Compared with the PTE model group, DNase I reduced circulating MPO-DNA complexes and attenuated TLR9/phospho-p65 signaling, while Fer-1 more prominently restored GPX4 expression. We interpret these results cautiously. Rather than claiming definitive causality, we now state that these findings provide **additional pharmacological support consistent with** the participation of NETs-associated signaling and ferroptosis-related injury processes in the observed phenotype.

This revised interpretation is intentionally more conservative and aligned with the level of evidence available.

### 3. Please provide tissue-level validation of key mechanisms and direct visualization of NETs within pulmonary thrombi.

**Response:**
Response:

We fully agree that direct tissue-level validation, including immunofluorescence-based visualization of NETs within pulmonary thrombi and in situ assessment of ferroptosis markers, would substantially strengthen the mechanistic conclusions.

At the same time, we would like to clarify that the primary focus of the present study was to characterize the dynamic changes in the systemic thrombo-inflammatory microenvironment using clinically accessible samples (i.e., peripheral blood), with an emphasis on temporal evolution and potential translational relevance. Therefore, our experimental design prioritized longitudinal whole-blood transcriptomic profiling, circulating biomarkers, and functional cardiopulmonary readouts, rather than tissue-resolved histological analysis.

Due to current technical limitations in obtaining well-validated antibodies for rabbit lung tissue, we were unable to perform tissue-level immunofluorescence in this revision. If deemed necessary by the Editor, we will make every effort to include such validation in a subsequent revision.

Importantly, we recognize that this design does not allow direct confirmation of tissue-level events such as NET formation within thrombi or ferroptotic cell death in situ. Accordingly, we have revised the manuscript throughout to avoid overinterpretation of these processes. We now frame our findings as evidence of NETs-associated and ferroptosis-related signaling programs at the systemic level, rather than definitive proof of tissue-level mechanisms.

To partially strengthen mechanistic support, we added an independent pharmacological validation experiment (Figure 7), in which DNase I and ferrostatin-1 (Fer-1) were used to target NETs-associated activity and ferroptosis-related processes, respectively. These data provide orthogonal, pharmacological support consistent with the involvement of these pathways, while still not replacing direct tissue-level validation.

We have explicitly acknowledged the absence of tissue-level validation as a limitation in the revised Discussion and identify this as an important direction for future studies. This study is therefore intended to complement, rather than replace, tissue-resolved mechanistic investigations.

We have therefore added clear limitation statements in the Discussion and Conclusion emphasizing that direct visualization of NETs in pulmonary thrombi and direct tissue-level assessment of ferroptosis remain important next steps for future work.**If deemed necessary, future revision or studies will incorporate tissue-level visualization of NETs within pulmonary thrombi to further confirm these mechanisms.**

### 4. MPO is not a specific marker for NETosis.

**Response:**
Response: We agree and revised the manuscript accordingly. In the time-course analysis, we emphasize MPO-DNA rather than MPO alone when discussing NETs-related changes. Importantly, MPO-DNA complexes have been widely used as a surrogate marker for NET formation and can be quantitatively measured by ELISA. Previous studies have demonstrated that MPO-DNA levels correlate well with the proportion of neutrophils undergoing NETosis, and this approach is considered one of the most specific and objective methods currently available for monitoring NET formation in biological samples (e.g., Clin Chim Acta, 2016).

In the new mechanistic validation section, we present the NETs assay as supportive pharmacological evidence and avoid claiming that it constitutes direct tissue-level proof of NETosis. We also tempered the mechanistic interpretation throughout the revised manuscript.

### 5. Addition of pro/anti-inflammatory marker detection would strengthen the manuscript.

**Response:**
We agree that broader inflammatory profiling would further strengthen the study. In the current revision, we prioritized the central concern raised by the editor and reviewers—namely, the need for stronger mechanistic support and more cautious interpretation. For this reason, we focused our new experimental effort on the added DNase I/Fer-1 pharmacological validation experiment centered on the NETs–TLR9–NF-kB/GPX4 pathway.

At the same time, we have moderated our claims throughout the manuscript and now identify more comprehensive inflammatory profiling as an important direction for future investigation.

### 6. Disclose all data points and mention the number of experiments done for each analysis in the figure legends.

**Response:**
We have revised the figure legends throughout the manuscript to specify the number of biologically independent animals used for each analysis (Figures 1–7). In addition, for the newly added quantitative validation experiment (Figure 7), all individual data points are shown explicitly. We also standardized the figure legends to clarify the meaning of abbreviations, the statistical tests used, and the sample sizes, thereby improving transparency and readability. These revisions are reflected in the updated figure legends of the revised manuscript.

### 7. Did the authors use iron-chelating agents as therapy in this model?

**Response:**
We did not use an iron-chelating agent in the present study. Instead, in the revised manuscript we added **ferrostatin-1 (Fer-1)** as a pharmacological ferroptosis inhibitor in the new validation experiment in order to test whether ferroptosis-related injury signaling may function downstream of NETs-associated activity. We have clarified this rationale in both the revised manuscript and the present response.

### 8. All transcriptomic analyses are based on whole blood, which limits biological specificity.

**Response:**
We agree completely that whole-blood transcriptomics has limited biological specificity and does not allow precise attribution of observed transcriptional changes to individual cell populations such as neutrophils, monocytes, or endothelial cells.

In response to this concern, we made two important revisions. First, we now explicitly acknowledge this limitation in the Discussion and state that future studies incorporating tissue-level assays and single-cell approaches will be required to resolve the cellular origin of these signals. Second, and importantly, we revised the wording throughout the manuscript to avoid attributing whole-blood transcriptional changes directly to neutrophils as a cell-intrinsic program. Expressions implying cell-specific regulation were replaced with more cautious terms such as **“neutrophil-associated,” “granule-related,”** or **“whole-blood signatures consistent with neutrophil activation.”**

We would also like to clarify that the primary purpose of the present transcriptomic analysis was to characterize the **temporal evolution of systemic thrombo-inflammatory changes** and to identify candidate circulating biomarkers with translational relevance, rather than to construct a cell-resolved atlas of the PTE microenvironment. We believe that whole-blood RNA-seq is appropriate for this biomarker-oriented and longitudinal objective, but we now clearly state that it cannot establish cell-type-specific transcriptional regulation.

### 9. Therapeutic intervention (rt-PA ± DNase I): clarification of DNase I administration, statistics, and need for a DNase I-only group.

**Response:**
We appreciate this important comment and have revised both the manuscript and the interpretation accordingly.

First, we corrected and standardized the description of DNase I administration to ensure consistency across the Abstract, Methods, Results, and figure legends. In the revised manuscript, DNase I is consistently described as **intravenous** administration.

Second, we revised the statistical description of the intervention experiment. In the revised manuscript, the **absolute pre-treatment and post-treatment values** are now reported alongside the derived improvement rates, and the statistical methods used for between-group comparisons are clarified in the Statistical Analysis section. We also clarified that the intervention results should be interpreted with caution given the sample size and number of endpoints assessed.

Third, we agree that inclusion of a **DNase I-alone** arm would have strengthened interpretation of whether the observed benefit is additive, synergistic, or partly independent of thrombolysis. However, the primary purpose of the intervention experiment in the current study was specifically to test whether DNase I could **enhance the efficacy of rt-PA** at the biologically selected day-7 window, rather than to evaluate DNase I monotherapy as a stand-alone thrombolytic strategy. We therefore focused the study design on **rt-PA alone versus rt-PA + DNase I**.

At the same time, we fully acknowledge that the absence of a DNase I-only group limits interpretation of additivity versus synergy, and we now state this explicitly as a limitation in the revised Discussion.

### 10. Introduction and Discussion enhancements.

**Response:**
We agree with these suggestions and revised the Introduction and Discussion accordingly. In the Introduction, we added a concise explanation of NETs, ferroptosis, and their potential biological connection to improve accessibility for readers. In the Discussion, we added a clearer comparison of the advantages and limitations of the rabbit autologous thrombus PTE model relative to alternative in vivo models. In addition, speculative statements—particularly those concerning self-reinforcing inflammatory loops and mechanistic interactions—have been clearly reframed as **hypotheses or interpretations**, rather than conclusions.

### 11. Protein-level validation and interpretation beyond RNA-based assays.

**Response:**
We appreciate the concern that qRT-PCR validation alone does not fully address the limitations of RNA-based inference, particularly in the context of terminally differentiated neutrophils, where transcriptome–proteome correlation may be limited.

To address this concern more directly, the revised manuscript now interprets the RNA-seq and qRT-PCR findings together with **orthogonal protein-level data**, including ELISA-based measurements of circulating MPO-DNA, TLR9, phospho-p65, total p65, GPX4, and selected NETs/iron-related markers. We have revised the text to clarify that these protein-based measurements are intended to provide additional supportive evidence and should not be viewed as stand-alone proof of cell-specific molecular mechanisms. We also now explicitly discuss the transcriptome–proteome limitation in neutrophils in the revised Discussion.

We sincerely thank the Editor and Reviewers again for the thoughtful and constructive feedback. We believe that the manuscript has been substantially improved through these revisions. Importantly, the revised version now presents the study as a **carefully framed longitudinal and pharmacologically supported preclinical investigation**, with conclusions that are more closely aligned with the available evidence. We hope that the revised manuscript will now be suitable for further consideration.

Sincerely,
Jialun Chen
